# AsaruSim: a single-cell and spatial RNA-Seq Nanopore long-reads simulation workflow

**DOI:** 10.1093/bioinformatics/btaf087

**Published:** 2025-02-22

**Authors:** Ali Hamraoui, Laurent Jourdren, Morgane Thomas-Chollier

**Affiliations:** GenomiqueENS, Institut de Biologie de l’ENS (IBENS), Département de biologie, École normale supérieure, CNRS, INSERM, Université PSL, Paris 75005, France; Group Bacterial Infection, Response & Dynamics, Institut de biologie de l’ENS (IBENS), École normale supérieure, CNRS, INSERM, Université PSL, Paris 75005, France; GenomiqueENS, Institut de Biologie de l’ENS (IBENS), Département de biologie, École normale supérieure, CNRS, INSERM, Université PSL, Paris 75005, France; GenomiqueENS, Institut de Biologie de l’ENS (IBENS), Département de biologie, École normale supérieure, CNRS, INSERM, Université PSL, Paris 75005, France; Group Bacterial Infection, Response & Dynamics, Institut de biologie de l’ENS (IBENS), École normale supérieure, CNRS, INSERM, Université PSL, Paris 75005, France

## Abstract

**Motivation:**

The combination of long-read sequencing technologies like Oxford Nanopore with single-cell RNA sequencing (scRNAseq) assays enables the detailed exploration of transcriptomic complexity, including isoform detection and quantification, by capturing full-length cDNAs. However, challenges remain, including the lack of advanced simulation tools that can effectively mimic the unique complexities of scRNAseq long-read datasets. Such tools are essential for the evaluation and optimization of isoform detection methods dedicated to single-cell long-read studies.

**Results:**

We developed AsaruSim, a workflow that simulates synthetic single-cell long-read Nanopore datasets, closely mimicking real experimental data. AsaruSim employs a multi-step process that includes the creation of a synthetic count matrix, generation of perfect reads, optional PCR amplification, introduction of sequencing errors, and comprehensive quality control reporting. Applied to a dataset of human peripheral blood mononuclear cells, AsaruSim accurately reproduced experimental read characteristics.

**Availability and implementation:**

The source code and full documentation are available at https://github.com/GenomiqueENS/AsaruSim.

## 1 Introduction

Single-cell RNA sequencing (scRNAseq) technologies have revolutionized our understanding of cell biology, providing high-resolution insights into Eukaryote cellular heterogeneity. Still, studying the heterogeneity at the level of isoforms and structural variations is currently limited. Traditional short-read sequencing coupled with single-cell technologies (commonly droplet-based scRNA-seq protocols such as 10X Genomics) are not suitable for studying full-length cDNAs, because they require RNA/cDNA fragmentation, often resulting in the loss of information regarding the complete exonic structure (Arzalluz-Luque and Conesa 2018). Combining long-read sequencing, such as Oxford Nanopore or Pacbio, with single-cell technologies has enabled addressing this challenge (Arzalluz-Luque and Conesa 2018). Despite its advantages, the quality of Nanopore sequencing used to be impacted by higher error rates compared to short-read technologies, thus negatively impacting the detection of cell barcodes (CBs) and unique molecular identifiers (UMIs) (Karst *et al*. 2021). Yet, these elements are critical for attributing reads to their original cells, and for the accurate characterization and quantification of isoforms. That is why a hybrid approach, coupling long-read and short-read technologies, used to be necessary for a reliable assignment of CBs and UMIs (Lebrigand *et al.* 2020). Recently, the accuracy of Nanopore reads has been drastically improved [95%–99% with the R10.3 flow cells (Dippenaar *et al*. 2022)], paving the way to untie long-read from short-read approaches in single-cell studies. Recently released bioinformatics methods, including scNapBar (Wang *et al.* 2021), FLAMES (Tian *et al.* 2021), BLAZE (You *et al.* 2023), Sicelore 2.1 (Lebrigand *et al.* 2020), Sockeye (https://github.com/nanoporetech/sockeye), and scNanoGPS (Shiau *et al.* 2023), have been developed to detect CBs and/or UMIs without using companion short-read data (referred to as Nanopore-only methods). These advances have the potential to reduce both the cost and the amount of work traditionally associated with hybrid sequencing computational workflows.

In the context of these developments, evaluating Nanopore-only methods for processing single-cell long-read datasets remains challenging. Most of the methods currently available are benchmarked against short-read datasets; this approach is not devoid of biases and is therefore considered to be an imperfect gold standard (Ziegenhain *et al.* 2022, Sun *et al.* 2024). One solution lies in the use of simulated datasets, which can mimic real experimental outcomes without the same biases as empirical methods. Simulated data provide a known ground truth—true CBs and true UMIs. This ground truth can be exploited by method developers in various ways, such as tuning method parameters, validating results, benchmarking novel tools against existing methods, and highlighting their performance across a wide range of scenarios. Besides, the focus of most long-read scRNA-seq and spatial methods is to identify alternative splicing events and differentially expressed isoforms (DEI) between cell types or cell states (Joglekar *et al.* 2023). Assessing the performance of these methods is also challenging because the ground truth is typically not known, and simulating random reads without any biological insight does not address this issue. One solution to this issue is to use instead simulated datasets, in which the ground truth (e.g. DEI, Fold change, batch effect) is known.

To date, no existing workflow has been designed with the specific purpose of simulating single-cell or spatial RNAseq long-read data, especially with biological insights. A general workflow for long-read transcriptomic datasets, TKSM (Karaoğlanoğlu *et al.* 2024), comprises some modules that enable users to assemble a pipeline for scRNAseq, but it is not primarily intended for single-cell applications. Current scRNAseq counts simulation tools [such as SPARSim (Baruzzo *et al.* 2020) or ZINB-WaVE (Risso *et al.* 2018)] generate only a synthetic single-cell count matrix. The bottleneck lies in the generation of simulated raw reads. It is notable that some studies on single-cell long-read methods, such as those described in Wang *et al.* (2021) and You *et al.* (2023), have employed simulated data. As part of these studies, individual tools (e.g. SLSim; https://github.com/youyupei/SLSim) have been developed to generate artificial template sequences with random cDNA, and simulators such as Badread (Wick 2019) or NanoSim (Yang *et al.* 2017) are employed to introduce sequencing errors based on a predefined error model. While such tools can effectively be used to benchmark the accuracy of CB assignment algorithms, it does not account for the complexities of estimating a realistic complete single-cell long-read dataset. Such complexities include polymerase chain reaction (PCR) biases and artifacts, sparsity, variability, and heterogeneity—characteristics intrinsic to single-cell and spatial data. Comprehensive simulation would allow for broader and more precise benchmarking of the performance of single-cell long-read bioinformatics tools.

To address this gap, we have developed AsaruSim, a workflow that simulates single-cell long-read Nanopore data. This workflow aims to generate a gold standard dataset for the objective assessment and optimization of single-cell long-read methods. The development of such a simulator alleviates the bottleneck in generating diverse *in silico* datasets by leveraging parameters derived from real-world datasets. This capability enables the assessment of method performance across different scenarios and refines pre-processing and analysis methods for handling the unique complexities of long-read data at the single-cell level.

## 2 Materials and methods

**Figure 1.**
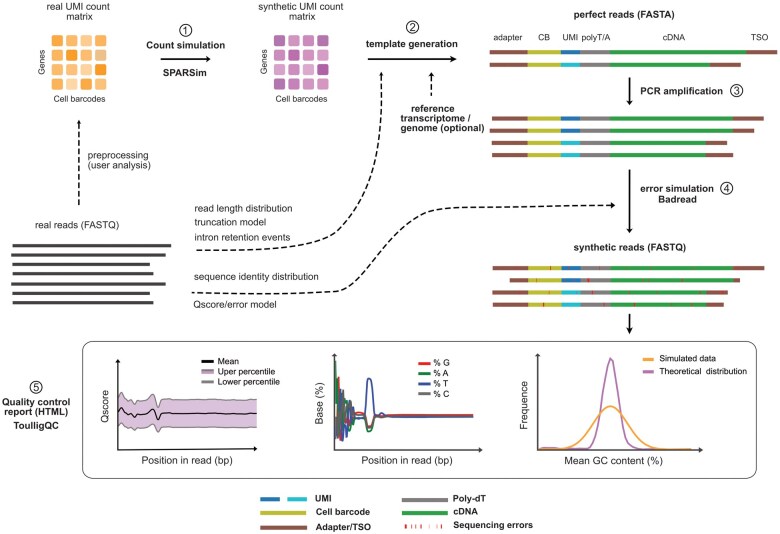
Summary of the AsaruSim workflow. It takes as input a real UMI count matrix and (1) trains the count simulator SPARSim to generate the corresponding synthetic UMI count matrix, serving as ground truth. It then (2) generates perfect reads (FASTA file) based on this synthetic UMI count matrix and a reference transcriptome. (3) It can optionally simulate bias introduced by PCR cycles. (4) It generates more realistic synthetic reads from the previous read templates (perfect or post-PCR) using a Badread simulator with a pre-trained error model on real Nanopore reads. (5) It outputs an HTML report presenting quality control plots that enable the user to assess the simulated reads, before using them to evaluate tools dedicated to analyze scRNAseq long-read data.

AsaruSim mimics real data by first generating realistic UMI counts using SPARSSim (Baruzzo *et al.* 2020), and then simulating realistic Nanopore reads using Badread (Wick 2019). Five major steps are implemented (Fig. 1).

### 2.1 Synthetic UMI count matrix

AsaruSim takes as input a feature-by-cell (gene/cell or isoform/cell) UMI count matrix (.CSV), which may be derived from an existing single-cell short- or long-read preprocessed run, or from a count simulator tool. The R SPARSim library (Baruzzo *et al.* 2020) is used to estimate the count simulation parameters from the provided UMI count matrix and generate the corresponding synthetic count matrices, taking advantage of its ability to support various input parameters. AsaruSim also enables the user to input their own count simulation parameters, or alternatively, to select them from a predefined set of parameters stored in the SPARSim database.

### 2.2 Perfect raw reads generation

This step is an original Python script. AsaruSim generates synthetic reads based on the synthetic count matrix. The retro-engineering of reads is achieved by generating a corresponding number of random UMI sequences for each feature (gene or isoform). The final construction corresponds to a 10X Genomics coupled with Nanopore sequencing library (Lebrigand *et al.* 2020): an adaptor sequence composed of 10× and Nanopore adaptors, a CB, UMI sequences at the same frequencies as in the synthetic count matrix, a 20-bp oligo(dT), the feature-corresponding cDNA sequence from the reference transcriptome, and a template switch oligo (TSO) at the end. When a gene expression matrix is provided, a realistic read length distribution is achieved by selecting a random transcript of the corresponding gene, with a prior probability in favor of short-length cDNA (Supplementary Note Sa). An optional step can be performed to mimick unspliced reads by retaining introns (Supplementary Note Sb). In real data, reads are not always full-length as cDNA can be truncated. Here, each generated cDNA is thus truncated based on an empirically derived truncation probability distribution, estimated by mapping a random subset of real reads to the reference transcriptome using Minimap2 (Supplementary Fig. S6a and b), as described in Prjibelski *et al.* (2023). At the end, generated reads are randomly oriented, with each synthetic read having an equal probability of being oriented in the original strand or the reverse strand. These final sequences are named “perfect reads” as they exactly correspond to the introduced elements (CB, UMI, cDNA…) without the addition of sequencing errors.

### 2.3 Mimicking PCR amplification bias (optional)

The perfect reads are duplicated through artificial multiple PCR cycles by an original Python script reimplemented from Sarkar *et al.* (2019) and Orabi *et al.* (2019) with several optimizations to improve speed and memory usage. This enables us to take into account the bias of amplification introduced during library constructions (Bolisetty *et al.* 2015). At each cycle, a synthetic read has a certain probability of being successfully replicated. The efficiency rate of duplication is fixed by the user (default *P*_dup_ = 0.9). Then, each nucleotide in the duplicated read has a probability of being mutated during the process. The error rate is also fixed by the user (default *P*_error_ = 3.5e−05). From this resulting artificial PCR product, a random subset of reads is finally selected to mimic the experimental protocol where only a subset of the sample is used for the sequencing step.

### 2.4 Introduction of sequencing errors in the reads

The perfect reads or post-PCR reads are used as a template for Badread error simulation, which simulates Nanopore sequencing errors and assigns per-base quality scores based on pre-trained error models and sequence identity with the reference genome. AsaruSim allows the user to (i) provide a personal pre-trained model, (ii) provide a real FASTQ read file to internally train a new model, or (iii) choose a pre-trained model within the Badread database. To approximate the observed sequence identity distribution in the experimental data, we align the real FASTQ read to the reference genome using Minimap2 (Li 2018), then calculate a sequence identity for each alignment from the Minimap2 output, with three possible identity models including or excluding gaps. A beta distribution is then fitted to the identity value to estimate the distribution parameters (Supplementary Note Sc).

### 2.5 Report

Finally, AsaruSim generates an HTML report presenting quality control plots obtained by analyzing the final FASTQ read files with ToulligQC (https://github.com/GenomiqueENS/toulligQC). This report aims to make sure the simulated data correspond to the expectations of the user before using them with tools dedicated to analyze scRNAseq long-read data.

AsaruSim is implemented in Nextflow (Di Tommaso *et al*. 2017) under GPL 3 license to allow a flexible and easily customizable workflow execution, computational reproducibility, and traceability (Supplementary Note Sd). To ensure numerical stability and easier installation, it also uses Docker (Merkel 2014) containerization technology.

## 3 Results

We developed AsaruSim to produce artificial Nanopore scRNAseq data that resembles a real experiment in terms of biological insights.

As a use case, we used a public dataset of human peripheral blood mononuclear cells (https://www.10xgenomics.com/datasets/5k-human-pbmcs-3-v3-1-chromium-controller-3-1-standard) as reference data. We downloaded the count matrix and used it as input to AsaruSim. From the 5000 cells initially present in the original matrix, we selected three cell types (CD8+T, CD4+T, and B cells) resulting in 1090 cells then used as a template to simulate the synthetic UMI count matrix (Step 1). Next, we simulated 20 million perfect reads (FASTA) (Step 2) with 10 PCR cycles (Step 3). We downloaded a subset of 1 million original FASTQ raw reads to generate the error model for Badread and then introduced errors to generate the synthetic reads (FASTQ) (Step 4). The quality control report is finally generated (Step 5, Supplementary Note Se).

We compared the properties of the simulated data to the experimental data. Both datasets showed similar (i) read length distribution and transcript coverage, (ii) number of mismatches and insertions/deletions in reads aligned to the 10× adapter sequence using VSEARCH (Rognes e*t al*. 2016) (Supplementary Note Se).

Next, we pre-processed the simulated raw reads using the Sockeye pipeline (https://github.com/nanoporetech/sockeye), and both experimental and simulated matrices were processed using Seurat v5 (Hao *et al*. 2024). The correlation of the average log fold change for cell type markers between real and simulated data shows a Pearson’s correlation coefficient *r* = 0.84 and the integration of both datasets shows a miLISI = 1.6, demonstrating a good agreement in gene expression between the real and simulated datasets (Supplementary Note Se).

When compared with TKSM (Karaoğlanoğlu *et al.* 2024), AsaruSim outperforms TKSM in terms of features specific to single-cell applications, similarity between real and simulated data, and computing efficiency (Supplementary Note Sf).

## 4 Conclusion

We presented a comprehensive workflow for simulating single-cell Nanopore data from the matrix to the sequence level, to create custom gold standard datasets. Potential applications include generating reads with differential gene expression or DEI between cell groups, as well as simulating known fold changes or batch effects, to assess and optimize single-cell long-read methods. AsaruSim offers a variety of configuration options to allow for flexible input and design.

Currently, AsaruSim generates data compatible with the 10X Genomics 3ʹ and spatial protocols. We plan to expand AsaruSim to accommodate additional single-cell techniques and protocols and support for PacBio sequencing.

## Supplementary Material

btaf087_Supplementary_Data

## Data Availability

All code used in this article is available at https://github.com/alihamraoui/AsaruSim_Application_Note. The data are accessible on Zenodo under DOI: 10.5281/zenodo.12731408.

## References

[btaf087-B1] Arzalluz-Luque Á , Conesa A. Single-cell RNAseq for the study of isoforms—how is that possible? Genome Biol 2018;19:1–19. 30097058 10.1186/s13059-018-1496-z PMC6085759

[btaf087-B2] Baruzzo G , Patuzzi I, Di Camillo B. SPARSim single cell: a count data simulator for scRNA-Seq data. Bioinformatics 2020;36:1468–75. 31598633 10.1093/bioinformatics/btz752

[btaf087-B3] Bolisetty MT , Rajadinakaran G, Graveley BR. Determining exon connectivity in complex mRNAs by nanopore sequencing. Genome Biol 2015;16:1–12. 26420219 10.1186/s13059-015-0777-z PMC4588896

[btaf087-B8638071] Di Tommaso P, , Chatzou M, , Floden EW et al Nextflow enables reproducible computational workflows. Nat Biotechnol 2017;35:316–9. 10.1038/nbt.3820. 28398311

[btaf087-B5942441] Dippenaar A, , Goossens SN, , Grobbelaar M et al Nanopore sequencing for mycobacterium tuberculosis: A critical review of the literature, new developments, and future opportunities. J Clin Microbiol 2022;60:e0064621. 10.1128/JCM.00646-21 34133895 PMC8769739

[btaf087-B4353256] Hao Y, , Stuart TIM, , Kowalski MH et al Dictionary learning for integrative, multimodal and scalable single-cell analysis. Nat Biotechnol 2024;42:293–304. 10.1038/s41587-023-01767-y 37231261 PMC10928517

[btaf087-B4] Joglekar A , Foord C, Jarroux J et al From words to complete phrases: insight into single-cell isoforms using short and long reads. Transcription 2023;14:92–104. 37314295 10.1080/21541264.2023.2213514 PMC10807471

[btaf087-B5] Karaoğlanoğlu F , Orabi B, Flannigan R et al TKSM: highly modular, user-customizable, and scalable transcriptomic sequencing long-read simulator. Bioinformatics 2024;40:2. 10.1093/bioinformatics/btae051 PMC10868325 38273664

[btaf087-B8336510] Karst SM, , Ziels RM, , Kirkegaard RH et al High-accuracy long-read amplicon sequences using unique molecular identifiers with nanopore or pacbio sequencing. Nat Methods 2021;18:165–9. 10.1038/s41592-020-01041-y 33432244

[btaf087-B6] Lebrigand K , Magnone V, Barbry P et al High throughput error corrected nanopore single cell transcriptome sequencing. Nat Commun 2020;11:4025. 32788667 10.1038/s41467-020-17800-6 PMC7423900

[btaf087-B7] Li H. Minimap2: pairwise alignment for nucleotide sequences. Bioinformatics 2018;34:3094–100. 29750242 10.1093/bioinformatics/bty191 PMC6137996

[btaf087-B8] Merkel D. Docker: lightweight Linux containers for consistent development and deployment. Linux J 2014:239:2.

[btaf087-B9] Orabi B , Erhan E, McConeghy B et al Alignment-free clustering of UMI tagged DNA molecules. Bioinformatics 2019;35:1829–36. 30351359 10.1093/bioinformatics/bty888

[btaf087-B10] Prjibelski AD , Alla M, Joglekar A et al Accurate isoform discovery with IsoQuant using long reads. Nat Biotechnol 2023;41:915–8. 36593406 10.1038/s41587-022-01565-y PMC10344776

[btaf087-B11] Risso D , Perraudeau F, Gribkova S et al A general and flexible method for signal extraction from single-cell RNA-Seq data. Nat Commun 2018;9:1–17. 29348443 10.1038/s41467-017-02554-5 PMC5773593

[btaf087-B9391928] Rognes T, , Flouri T, , Nichols BEN et al VSEARCH: A versatile open source tool for metagenomics. PeerJ 2016;4:e2584. 10.7717/peerj.2584 27781170 PMC5075697

[btaf087-B12] Sarkar H , Srivastava A, Patro R. Minnow: a principled framework for rapid simulation of dscRNA-Seq data at the read level. Bioinformatics 2019;35:i136–44. 31510649 10.1093/bioinformatics/btz351 PMC6612833

[btaf087-B13] Shiau C-K , Lu L, Kieser R et al High throughput single cell long-read sequencing analyses of same-cell genotypes and phenotypes in human tumors. Nat Commun 2023;14:4124. 37433798 10.1038/s41467-023-39813-7 PMC10336110

[btaf087-B14] Sun J , Philpott M, Loi D et al Correcting PCR amplification errors in unique molecular identifiers to generate accurate numbers of sequencing molecules. Nat Methods 2024;21:401–5. 38317008 10.1038/s41592-024-02168-y PMC10927542

[btaf087-B15] Tian L , Jabbari JS, Thijssen R et al Comprehensive characterization of single-cell full-length isoforms in human and mouse with long-read sequencing. Genome Biol 2021;22:310. 10.1186/s13059-021-02525-6 34763716 PMC8582192

[btaf087-B16] Wang Q , Boenigk S, Boehm V et al Single-cell transcriptome sequencing on the nanopore platform with ScNapBar. RNA 2021;27:763–70. 33906975 10.1261/rna.078154.120 PMC8208055

[btaf087-B17] Wick RR. Badread: simulation of error-prone long reads. JOSS 2019;4:1316.

[btaf087-B18] Yang C , Chu J, Warren RL et al NanoSim: nanopore sequence read simulator based on statistical characterization. Gigascience 2017;6:1–6. 10.1093/gigascience/gix010 PMC5530317 28327957

[btaf087-B19] You Y , Prawer YDJ, De Paoli-Iseppi R et al Identification of cell barcodes from long-read single-cell RNA-seq with BLAZE. Genome Biol 2023;24:66. 37024980 10.1186/s13059-023-02907-y PMC10077662

[btaf087-B20] Ziegenhain C , Hendriks G-J, Hagemann-Jensen M et al Molecular spikes: a gold standard for single-cell RNA counting. Nat Methods 2022;19:560–6. 35468967 10.1038/s41592-022-01446-x PMC9119855

