# Supplementary material for: AsaruSim: a single-cell and spatial RNA-Seq Nanopore long-reads simulation workflow
Source: Bioinformatics. 2025 Feb 22;41(3):btaf087. doi: 10.1093/bioinformatics/btaf087 (PMC11897429; doi:10.1093/bioinformatics/btaf087)
Supplement: btaf087_Supplementary_Data [file btaf087_supplementary_data.pdf]

AsaruSim: a single-cell and spatial RNA-Seq Nanopore long-reads simulation workflow

ALI HAMRAOUI[1,2] , LAURENT JOURDREN[1], MORGANE THOMAS-CHOLLIER[1,2]

1. GenomiqueENS, Institut de Biologie de l'ENS (IBENS), Département de biologie, École normale supérieure, CNRS, INSERM, Université PSL, 75005 Paris, France
2. Group Bacterial infection, response & dynamics, Institut de biologie de l'ENS (IBENS), École normale supérieure, CNRS, INSERM, Université PSL, 75005 Paris, France

Corresponding Author: mthomas@bio.ens.psl.eu

Supplementary Notes

a — Mimicking the real read length distribution (for use in step 2)

AsaruSim generates reads that correspond to a 10X Genomics coupled with Nanopore library construction (Figure 1). Regarding the cDNA sequences, the length of the reference annotation sequences may not mimic real datasets. Indeed, in the publicly available data generated with 10X Genomics devices coupled with Nanopore sequencing, it has been observed that the average read length does not exceed 1.2 kb (i.e. Tian et al. 2021; Shiau et al. 2023). Yet, many organisms (such as human or mouse) have an average cDNA length that exceeds this value; using the full length of the reference cDNA for the simulated data would thus not be realistic (Supplementary figure S1a).

If the user provides a gene expression matrix, a realistic read length distribution is achieved by selecting a random transcript of the corresponding gene, with a prior probability in favor of short length cDNA. To approximate the observed distribution of read lengths, we first identified the statistical distribution that best describes the real dataset. A number of candidate distributions (Weibull, gamma, log-norma) are fitted with the real data. Then, we calculated an Akaike information criteria AIC; $AIC = 2K - 2ln(L)$, where K is the number of reads and L is the log-likelihood estimated from the probability density function. Based on the comparison of the AIC values, we determined that the log-normal distribution best approximates the read length distribution (Supplementary figure S1b).

This log-normal distribution is then used to efficiently approximate the cDNA length distribution of the real dataset. We implemented a model-fitting approach so that users may fit their data by providing a subset of real reads (in FASTQ format, Figure 1). Then, the perfect reads (step 2) are generated by selecting a random transcript of the corresponding gene. When a gene-by-cell matrix is provided, the choice of this reference cDNA is achieved by using the estimated log-normal distribution as a probability function to give more chance

Distributed under a Creative Commons Attribution | CC-BY 4.0 International licence

to transcripts having the length that matches the real read length distribution (thus favoring short length transcripts).

An alternative input to the feature-by-cell UMI count matrix is the submission of a per barcode UMI count CSV file to AsaruSim. The user thus only controls the cell number and sequencing depth of synthetic data. In this scenario, the genes will be randomly selected from the reference transcriptome following the estimated read length probability function.

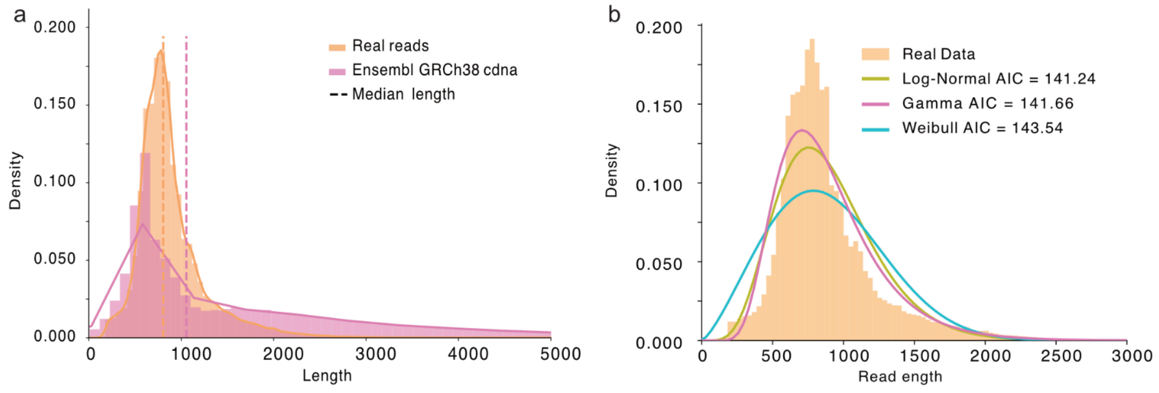

Figure S1. Read length distributions. a. Comparison between Ensembl cDNA (median 1055 pb) and real reads lengths (median = 806 bp). The real reads comprise the real cDNA sequence (median ~700 bp) and ~100 bp of various adaptor sequences. **b.** Fitting of theoretical distributions to real data. Different theoretical candidate distributions (Log-Normal, Gamma and Weibull) are fitted to the real read length distribution. The Akaike information criterion (AIC) shows that the Log-Normal distribution best fits the real read distribution**.**

b — Simulating unspliced reads and Intron retention events

Real single-cell, and especially single-nuclei, libraries contain a significant proportion of intronic reads—up to 20% in single-cell 3' human PBMC datasets (10x Genomics, Technical Note CG000376). These reads can originate from unprocessed pre-mRNA and intron-retained transcripts, although the latter typically occur at lower proportions. To simulate intron retention events, we use a Markov chain model to represent the transitional probabilities between spliced and retained intron states, based on the state of the preceding intron, as described in NanoSim (Hafezqorani et al., 2020). This model is generated by running the model_intron_retention.py module from NanoSim on real FASTQ reads. Additionally, to simulate unprocessed pre-mRNA, AsaruSim allows users to specify a fraction of reads that will remain unspliced; in this fraction, all introns within the transcript are retained. This feature is demonstrated in Supplementary note e.

c — Sequence identity estimation (for use in step 4)

Distributed under a Creative Commons Attribution | CC-BY 4.0 International licence

Badread uses the beta distribution to model the sequence identity distribution. In order to identify the optimal parameters (mean, standard deviation, and maximum) that best describe this beta distribution for our real data, a step search method was conducted throughout simulations of multiple subsets, each containing 100,000 reads simulated from the human reference transcriptome hg38. Briefly, in each step we compare the subset to the real data and rerun the simulation with one of the three parameters adjusted. To compare the subset to the real data, the 10X adapter sequence was aligned to the real and simulated reads using VSEARCH (T et al. 2016). The resulting sequence identities were then used to compute an MSE between the real and simulated reads:

$$MSE = \frac{1}{n}\sum_{i=1}^{n} (mis_{real,i} - mis_{sim,i})^2$$

as $mis_{real,i}$ and $mis_{sim,i}$ are the number of mismatches from real and simulated data. Further details of this simulation can be found in our GitHub repository (https://github.com/GenomiqueENS/AsaruSim).

Sequence identity can be computed in multiple ways from a sequence alignment. We provide three identity models : the most commonly used method is "Gap-Excluded Identity", which excludes all gapped positions from the alignment, calculated as:

$$\text{Identity} : \frac{Number\ of\ matches}{Number\ of\ matches + Number\ of\ mismatches}.$$

Alternatively, the "BLAST Identity" (Altschul et al., 1990) is defined as the number of matching bases divided by the total number of alignment positions. "Gap-Compressed Identity" treats consecutive gaps as a single difference. Even though Badread uses BLAST definition to define sequence identity, our simulation shows that using the "Gap-Excluded Identity" in our model best approximates the real sequence identity (Supplementary figure S2). That's why we set as default the Gap-Excluded Identity model.

Distributed under a Creative Commons Attribution | CC-BY 4.0 International licence

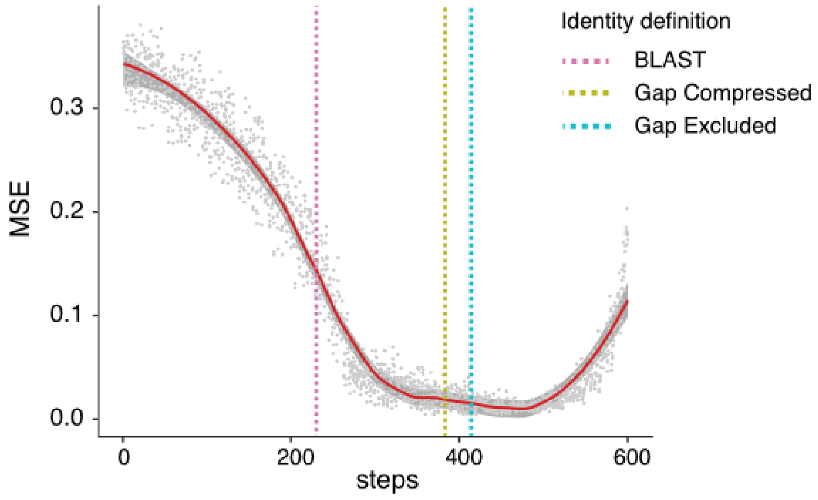

Figure S2. Parameter selection. Variation of mean square error (MSE) for sequence identity between real and simulated reads across different beta distribution parameters. To determine the optimal beta parameters that best approximate the sequence identity of real data, we conducted step search study through multiple simulations, adjusting one of the three parameters (mean, std, max) at each step. To measure the Mean Square Error (MSE) between real and simulated reads, we first aligned the adapter sequence to the real and simulated reads using VSEARCH (T et al. 2016). Then we used the resulting number of mismatches to calculate an MSE. Next, we plotted the parameters found with our fitted model using each definition of sequence identity (BLAST, Gap-compressed and Gap-excluded; vertical lines).

d — Nextflow implementation

AsaruSim is implemented in Nextflow to allow an easier installation and a robust processing and reproducibility. It allows flexible inputs; users may provide a UMI count matrix (.CSV), or a list of barcode counts (.CSV) or selection of the existing dataset from the SPARSim database. Additionally, users have the option to supply a reference raw read (.FASTQ) to estimate the error profile or choose from a wide range of predefined parameters. AsaruSim generates the simulated reads in FASTQ format and provides a comprehensive quality control report in HTML format (Supplementary Fig. S3).

Distributed under a Creative Commons Attribution | CC-BY 4.0 International licence

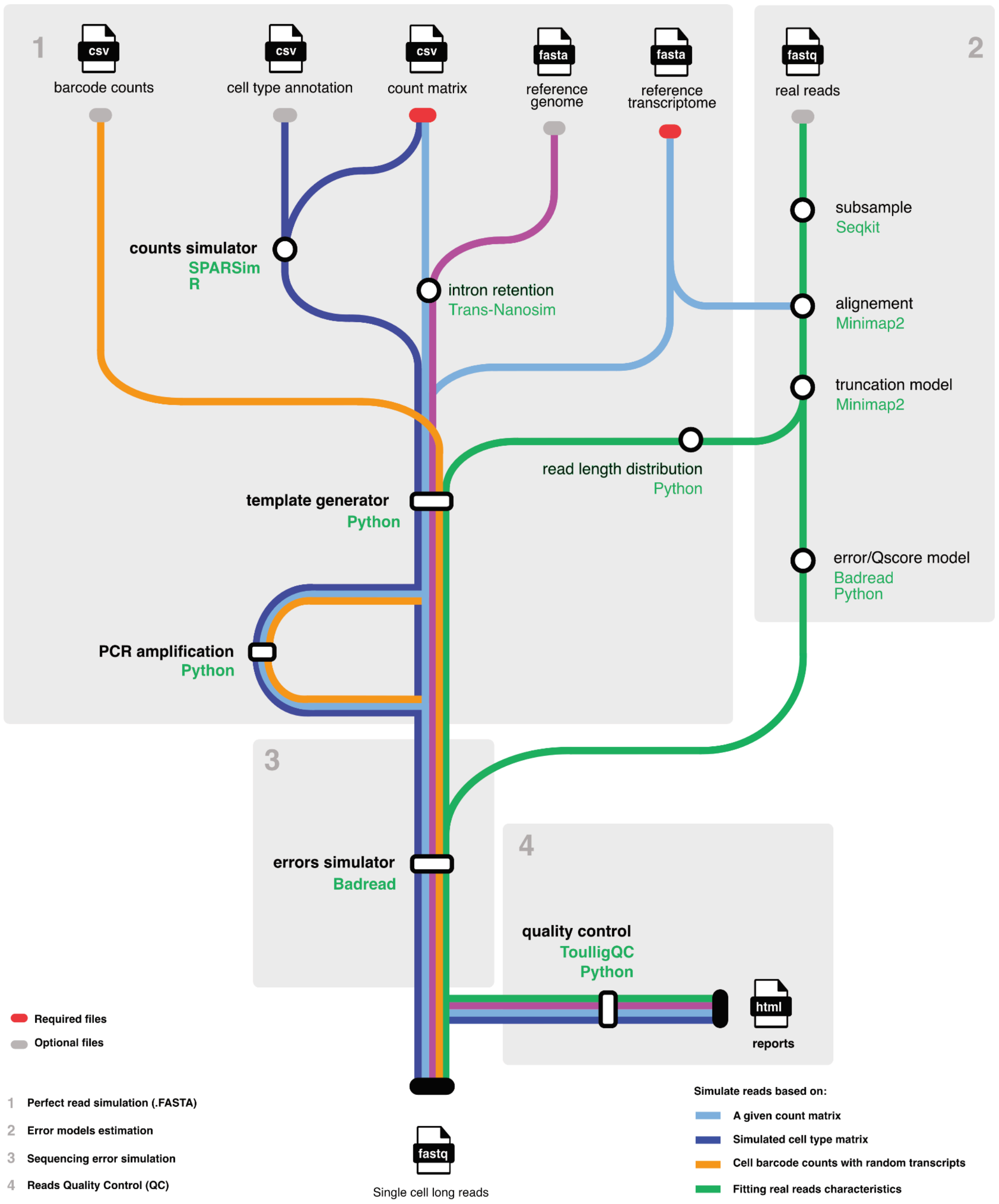

Figure S3. **Workflow schema.** Schematic representation of AsaruSim nextflow workflow showing the main 4 steps: 1- synthetic UMI count matrix, 2- perfect raw reads generation, 3- mimicking PCR amplification bias (optional), 4- introduction of sequencing errors in the reads, and 5- Quality control report.

e — Comparison of real *vs* simulated reads in the PBMC study case.

In this section, we compared the characteristics of our simulated data (Supplementary Figure S4) with the real single cell long read data used as reference. The PBMC dataset, jointly produced by 10X Genomics and Nanopore was downloaded from (« 5k Human PBMCs, 3' v3.1, Chromium Controller », s. d.).

Distributed under a Creative Commons Attribution | CC-BY 4.0 International licence

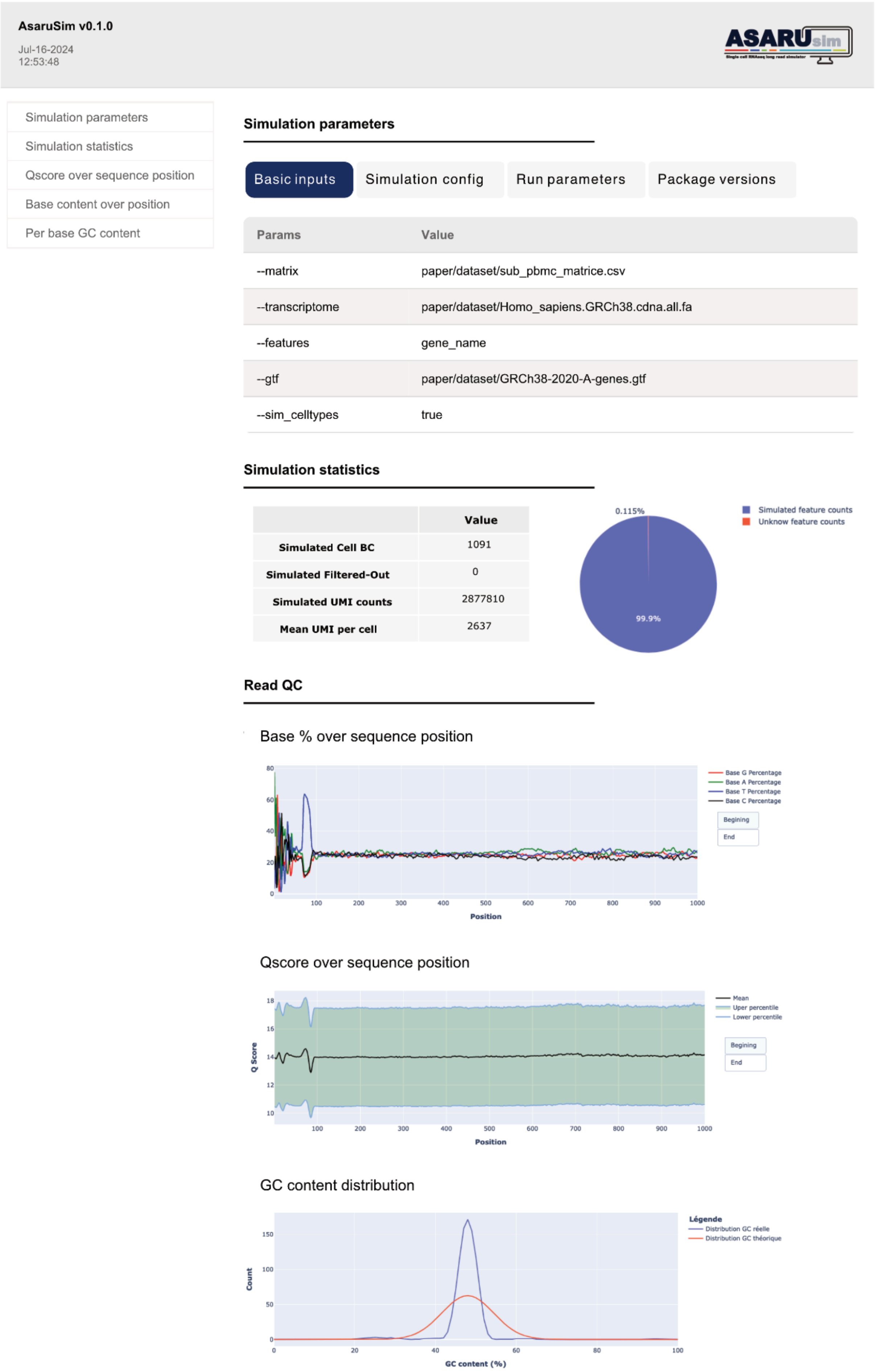

Distributed under a Creative Commons Attribution | CC-BY 4.0 International licence

Figure S4. Quality control report: A capture of the HTML report generated by AsaruSim. The report is divided into three sections: Simulation parameters section, includes files and parameters used for the simulation to ensure traceability. Simulation statistics section**,** provides simulation statistics, including the number of BCs and UMIs, as well as general statistics about the run. Read QC section, provides the median Qscores, the percentage of bases over read sequences, and the GC content percentage.

We first compared our simulated data with real data in terms of read length distribution, transcript coverage, number of mismatch and number of substitutions (Supplementary Figure S5). We observed similar properties between the simulated and real datasets. The distribution of read lengths was similar across the two datasets (Supplementary Figure S5a). After alignment to the human hg38 genome, both datasets show comparable gene body coverage (Supplementary Figure S5b) ; the post-alignment QC results using Picard toolkit (Broad Institute., 2019) on simulated data combining intron retention events and 7% of random unspliced transcripts, indicate similar proportions of bases aligning to intronic, UTR, and coding regions (Supplementary Figure S5c).

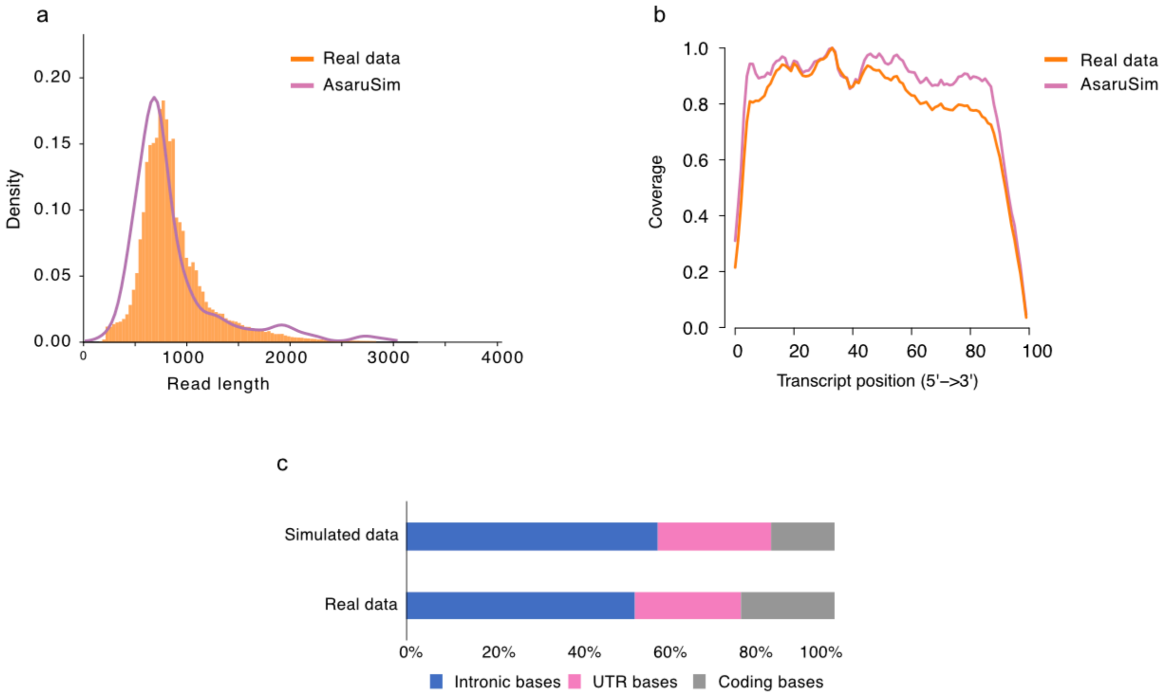

Figure S5. Comparison of real vs simulated reads. a. Read length distribution of the real and simulated reads. The distribution used for simulated cDNA size is obtained by fitting the read size distribution observed in the experimental data. **b.** Transcript body coverage plot for the simulated reads and real reads. This plot was generated with the Qualimap tool (Okonechnikov, Conesa, et García-Alcalde 2016) using as input the BAM file tagged by Sockeye workflow (nanoporetech, 2023). **c.** Post-alignment QC results compare the alignment of real and simulated datasets (combining intron

Distributed under a Creative Commons Attribution | CC-BY 4.0 International licence

retention events and 7% of random unspliced transcripts) to the human hg38 reference genome. Figure representing percent of bases aligned to intronic, UTR and coding regions.

After alignment to the 10X Genomics adapter sequence using VSEARCH (T et al. 2016)., the number of mismatches, indel errors and percentage of alignment identity (97%) revealed very similar between the real and simulated data (Supplementary Figure S6c, S6d). These results demonstrate the capability of AsaruSim to effectively simulate data that closely emulates real data.

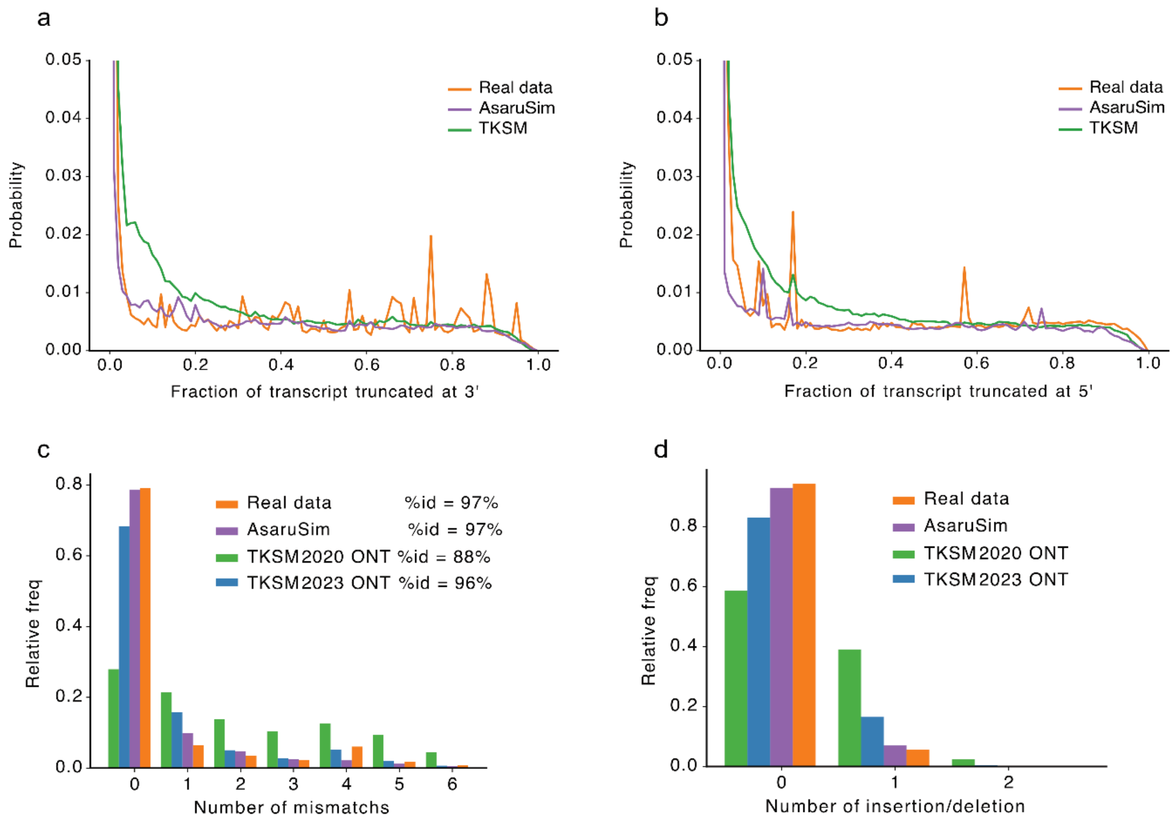

Figure S6. Comparison of AsaruSim vs TKSM simulated reads. a. Empirical 3' truncation probability distributions for real human PBMCs ONT data (10X, 2022) versus data simulated using either AsaruSim or TKSM. The distributions were estimated by mapping reads to the human hg38 reference transcriptome using Minimap2. **b.** Same as panel a, but for the empirical 5' truncation probability. **c.** Number of mismatches and percentage of alignment identity in reads aligned to 10X Genomics adapter sequence using VSEARCH (T et al. 2016), Two of Badread's recommended read identity parameters were tested with TKSM: the default parameters (for nanopore R9.4.1 chemistry) and the latest model (95,99,2.5), corresponding to the R10.4.1 chemistry. **d.** Number of gaps in reads aligned to 10X Genomics adapter sequence using VSEARCH.

We then preprocessed the simulated raw reads and the real reads in a similar way using the Sockeye workflow (nanoporetech, 2023), and performed downstream analyses of the

resulting gene count matrices with seurat V5. We compared the t-SNE visualisation of simulated and real data (Supplementary Figure S7a and S7c). miLISI measures the degree of mixing among datasets; the value of 1.6 shows a good closeness between the real and simulated data. Finally, we found a high correlation of the average log fold change for the cell types markers between real and simulated data (Pearson's r=0.84). We conclude that our simulation workflow is robust to simulate single-cell RNA-seq long reads with gene expression profiles closely resembling those of real datasets.

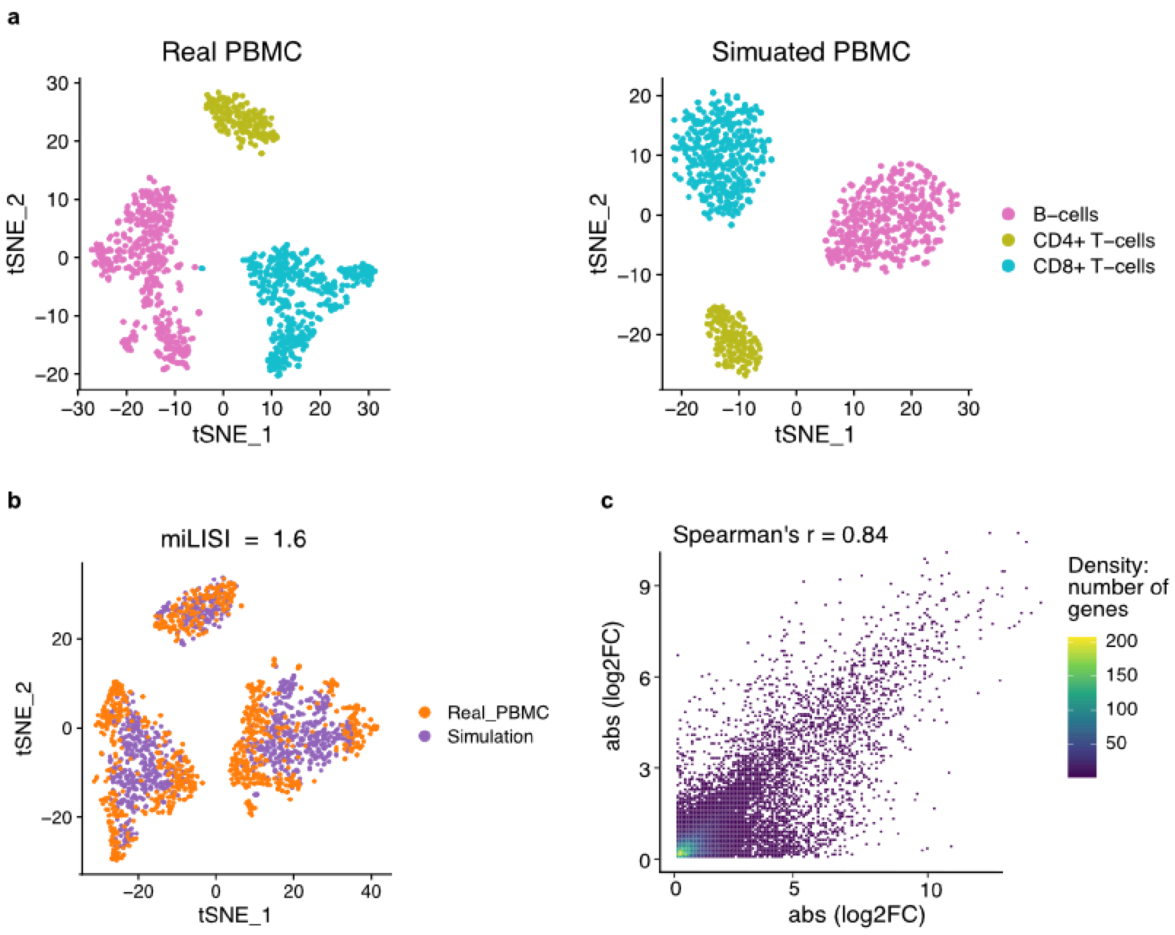

Figure S7. The t-SNE plots of real vs simulated gene expression count matrices. a. t-SNE representation based on the dataset of real PBMC cells (left) or AsaruSim simulated data (right). The matrices were processed using Seurat v5. For quality control purposes, genes expressed in less than three cells were discarded, and cells with gene counts more than 3,000 or fewer than 200 were filtered out. Then, cells were colored following the cell types annotation which was obtained using the singleR R package (Aran et al. 2019). **b.** Pairwise integration of two single cell datasets. The Seurat CCAIntegration algorithm was used for integration and the batch effect is removed using Seurat V5. The mean integration Local Inverse Simpson Index (miLISI) score was calculated using the LISI R package (Korsunsky et al. 2019). **c.** Density scatter plot shows the correlation of the absolute value of

Distributed under a Creative Commons Attribution | CC-BY 4.0 International licence

average log fold change (log2FC) for the cell types markers between real and simulated data. The log2FC values are computed using Seurat v5 FindMarker function.

f — Comparison between AsaruSim and TKSM.

TKSM (Karaoğlanoğlu et al. 2024) is a transcriptomic sequencing long-read simulator providing modules that users can assemble into a pipeline, according to the sequencing design of the real dataset. Both AsaruSim and TKSM benefit from a workflow manager (Supplementary Table S1). In terms of features, both AsaruSim and TKSM take into account PCR cycles, Error and Qscore modeling, and truncation of reads modeling. TKSM supports gene/isoform fusion. In contrast to TKSM, AsaruSim provides unique features including read identity modeling, unspliced and novel transcripts simulation, as well as an HTML report to control the quality of the simulated dataset. Contrary to TKSM, AsaruSim does not take as input a FASTQ file of real reads, which users may not have, especially for public datasets that are available as already-processed count matrices. That's why AsaruSim takes as input a count matrix (gene or isoform). A distinct feature of AsaruSim is its capability of simulating counts (step 1) with user's personal simulation parameters, based on SPARSim (Baruzzo, Patuzzi, et Di Camillo 2020). For sake of comparison, Table S1 includes SLSim (You et al. 2023), which simulates basic reads, including only the Error and Qscore modeling.

Distributed under a Creative Commons Attribution | CC-BY 4.0 International licence

	AsaruSim	TKSM	SLSim
Programming language	Python/R	C++/Python	Python
Workflow manager	Nextflow	Snakemake	X
Input	Gene/isoform counts matrix<br>CB counts<br>SPARSim presets	FASTQ	CB counts
Counts simulation	V	X	X
PCR simulation	V	V	X
Error and Qscore modeling	V	V	V
Read Identity modeling	V	X	X
Truncation modeling	V	V	X
Intron retention / unspliced transcripts	V	X	X
Gene/isoform fusion	X	V	X
Novel isoforms	V	X	X
Simulation QC report	V	X	X

Table S1. Comparison of AsaruSim versus TKSM and SLSim features.

We have adapted TKSM to build a pipeline customized to the sequencing construction used to produce the PBMC study dataset (available on our reproduction package: https://github.com/alihamraoui/AsaruSim_Application_Note). We then simulated data with AsaruSim and TKSM, providing as input the FASTQ reads to TKSM and the count matrix to AsaruSim. We compared features common to both tools: Error and Qscore modeling as well as the truncation modelling (Supplementary Figure S6). The truncation profile of AsaruSim is more similar to the truncation profile of the real dataset than TKSM's profile, in particular for the truncation probability distribution values, where TKSM tends to over-truncate read sequences. This observation applies for both 3' and 5' truncation (Supplementary Figure S6a,b). Regarding the error modelling, we aligned the reads to the 10X Genomics adapter sequence to calculate the number of mismatches, indel errors and percentage of alignment identity (Supplementary Figure S6c,d). The results obtained with AsaruSim are consistently more similar to the real data than TKSM, even when modifying TKSM to incorporate Badread's read identity parameters for the latest R10.4.1 chemistry. These results demonstrate the higher capability of AsaruSim to simulate data that more accurately emulates real sequencing data.

Distributed under a Creative Commons Attribution | CC-BY 4.0 International licence

Last, we compared the computing efficiency of both tools (Supplementary Table S2). The runtime of AsaruSim is significantly lower than TKSM, thanks to a more efficient parallelisation of Badread. The peak memory is also lower for AsaruSim.

We compared the computational efficiency of both tools (Supplementary Table S2). AsaruSim demonstrates significantly lower runtime compared to TKSM, attributed to its more efficient parallelization of Badread. Additionally, AsaruSim requires less peak memory, further highlighting its computational advantages.

	Running time	Peak RAM (Gb)
AsaruSim	0h 44min 45s	26.7
TKSM	5h 45min 12s	33.9

Table S2. Comparison of AsaruSim and TKSM runtime and peak memory usage. Runtime and peak memory were measured using the Linux time command with the -v option during the simulation of 5,000 cells with 5 PCR cycles, totaling 5 million reads. Simulations were executed on the same machine (32 cores, 189 GB RAM) using 20 threads.

References

10x Genomics, 2021. Interpreting Intronic and Antisense Reads in 10x Genomics Single Cell Gene Expression Data. Technical Note, CG000376.

« 5k Human PBMCs, 3' v3.1, Chromium Controller ». s. d. 10x Genomics. Consulté le 21 mai 2024. https://www.10xgenomics.com/datasets/5k-human-pbmcs-3-v3-1-chromium-controller-3-1-standard.

Aran, Dvir, Agnieszka P. Looney, Leqian Liu, Esther Wu, Valerie Fong, Austin Hsu, Suzanna Chak, et al. 2019. « Reference-Based Analysis of Lung Single-Cell Sequencing Reveals a Transitional Profibrotic Macrophage ». *Nature Immunology* 20 (2): 163‑72. https://doi.org/10.1038/s41590-018-0276-y.

Baruzzo, Giacomo, Ilaria Patuzzi, et Barbara Di Camillo. 2020. « SPARSim Single Cell: A Count Data Simulator for scRNA-Seq Data ». *Bioinformatics* 36 (5): 1468‑75. https://doi.org/10.1093/bioinformatics/btz752.

Broad Institute. 2019. « Picard Toolkit ». GitHub repository. https://broadinstitute.github.io/picard/.

Karaoğlanoğlu, Fatih, Baraa Orabi, Ryan Flannigan, Cedric Chauve, et Faraz Hach. 2024. « TKSM: Highly Modular, User-Customizable, and Scalable Transcriptomic Sequencing Long-Read Simulator ». *Bioinformatics* 40 (2). https://doi.org/10.1093/bioinformatics/btae051.

Korsunsky, Ilya, Nghia Millard, Jean Fan, Kamil Slowikowski, Fan Zhang, Kevin Wei, Yuriy Baglaenko, Michael Brenner, Po-ru Loh, et Soumya Raychaudhuri. 2019. « Fast, Sensitive and Accurate Integration of Single-Cell Data with Harmony ». *Nature*

Distributed under a Creative Commons Attribution | CC-BY 4.0 International licence

Methods 16 (12): 1289‑96. https://doi.org/10.1038/s41592-019-0619-0.

« nanoporetech/sockeye ». (2022) 2023. Python. Oxford Nanopore Technologies. https://github.com/nanoporetech/sockeye.

Okonechnikov, Konstantin, Ana Conesa, et Fernando García-Alcalde. 2016. « Qualimap 2: Advanced Multi-Sample Quality Control for High-Throughput Sequencing Data ». *Bioinformatics* 32 (2): 292‑94. https://doi.org/10.1093/bioinformatics/btv566.

Shiau, Cheng-Kai, Lina Lu, Rachel Kieser, Kazutaka Fukumura, Timothy Pan, Hsiao-Yun Lin, Jie Yang, et al. 2023. « High Throughput Single Cell Long-Read Sequencing Analyses of Same-Cell Genotypes and Phenotypes in Human Tumors ». *Nature Communications* 14 (1): 4124. https://doi.org/10.1038/s41467-023-39813-7.

T, Rognes, Flouri T, Nichols B, Quince C, et Mahé F. 2016. « VSEARCH: A Versatile Open Source Tool for Metagenomics ». *PeerJ* 4 (octobre). https://doi.org/10.7717/peerj.2584.

Tian, Luyi, Jafar S. Jabbari, Rachel Thijssen, Quentin Gouil, Shanika L. Amarasinghe, Oliver Voogd, Hasaru Kariyawasam, et al. 2021. « Comprehensive characterization of single-cell full-length isoforms in human and mouse with long-read sequencing ». *Genome Biology* 22 (1): 310. https://doi.org/10.1186/s13059-021-02525-6.

You, Yupei, Yair D. J. Prawer, Ricardo De Paoli-Iseppi, Cameron P. J. Hunt, Clare L. Parish, Heejung Shim, et Michael B. Clark. 2023. « Identification of cell barcodes from long-read single-cell RNA-seq with BLAZE ». *Genome Biology* 24 (1): 66. https://doi.org/10.1186/s13059-023-02907-y.

Distributed under a Creative Commons Attribution | CC-BY 4.0 International licence
